# Gender-related power differences, beliefs and reactions towards people living with HIV/AIDS: an urban study in Nigeria

**DOI:** 10.1186/1471-2458-10-334

**Published:** 2010-06-12

**Authors:** Ngozi C Mbonu, Bart Van den Borne, Nanne K De Vries

**Affiliations:** 1 Department of Health Promotion, School of Public Health and Primary Care CAPHRI Faculty of Health, Medicine and Life Sciences, Maastricht University, PO Box 616, Maastricht 6200 MD, The Netherlands

## Abstract

**Background:**

Although there are an increasing number of studies on HIV-related stigma in Nigeria, very little research has focused on how power differences based on gender perpetuate the stigmatization of people living with HIV/AIDS (PLWHA) and how these gender differences affect the care that PLWHA receive in health care institutions. We explore gender-related beliefs and reactions of society, including health care professionals (HCPs), with regard to PLWHA, using Connell's theoretical framework of gender and power (1987). With Connell's structural theory of gender and power (financial inequality, authority and structure of social norms), we can describe gender differences in stigmatization of PLWHA.

**Method:**

We conducted in-depth semi-structured interviews, lasting 60 to 90 minutes, with 100 persons (40 members of the general public, 40 HCPs and 20 PLWHA) in Port Harcourt, Nigeria. The interviews were tape-recorded and transcribed verbatim. The Nvivo 7 computer package was used to analyze the data.

**Results:**

There are similarities and differences between the general public and HCPs towards PLWHA in gender-related beliefs and reactions. For instance, although association with promiscuity and power differences were commonly acknowledged in the different groups, there are differences in how these reactions are shown; such as HCPs asking the female PLWHA to inform their partners to ensure payment of hospital bills. Women with HIV/AIDS in particular are therefore in a disadvantaged position with regard to the care they receive.

**Conclusion:**

Despite the fact that men and women with HIV/AIDS suffer the same illness, clear disparities are apparent in the negative reaction women and men living with HIV/AIDS experience in society. We show that women's generally low status in society contributes to the extreme negative reactions to which female PLWHA are subject. The government should create policies aimed at reducing the power differences in family, society and health care systems, which would be important to decrease the gender-related differences in stigma experienced by PLWHA. Interventions should be directed at the prevailing societal norms through appropriate legislation and advocacy at grassroots level with the support of men to counter laws that put women in a disadvantaged position. Furthermore, development of a policy that encourages equality in access to health care for all patients with HIV/AIDS by applying the same conditions to both men and women in health care institutions is recommended. There is a need to protect women's rights through implementing support policies, including paying attention to gender in the training of HCPs.

## Background

Women and girls continue to be affected disproportionately by HIV in Sub-Saharan Africa [1]. In Nigeria, the HIV prevalence rate among females aged 15-24 years was 2.3% in 2008 while the prevalence rate among males aged 15-24 years was much lower at 0.8% [2]. According to a United Nation Human Development report on Nigeria, the burden of HIV infection in Nigeria is borne by young people, with more females affected than males [3]. This means that females may be more vulnerable to the medical and social consequences associated with living with HIV/AIDS, such as experiencing more negative reactions than their male counterparts in society.

Ever since stigmatization has been described as a discrediting attribute [4], researchers have emphasized the importance of conceptualizing stigma in terms of power differences that feed on existing inequalities in the society, such as those found in gender [5,6]. While sex can be defined as the biological distinction between men and women [7], Schur (p. 10) describes gender as "the socio cultural and psychological shaping, patterning and evaluation of male and female behaviour" [8]. Butler (p. 42) similarly noted that gender is a mechanism by which notions of masculinity and femininity are produced and naturalized [9]. It is important to understand how society, including the health care system, allows gender differences in behaviour towards PLWHA. In this context, theories founded on gender become critical.

Connell (1987) elaborated one of the most integrative theories of gender [10]. This theory is important because it allows for an understanding of the complex interplay between gender and power beyond the individual perspective. A central emphasis of this theory is that the analysis of gender involves a three-part structural model involving sexual division of labour (e.g. financial inequality), sexual division of power (e.g. authority), and the structure of affective attachments (e.g. social norms). These three structural models are the major elements of any gender order and operate with a logical complexity. Furthermore, these structural models exist at different levels (e.g. family, societal and institutional) and are maintained by social mechanisms. Connell's theory of gender and power has been shown previously to explain the gender effects in the spread of HIV/AIDS infection [11,12]. Sa and Larsen applied this theory in their study in Moshi, Tanzania, using gender inequality to explain women's risk of HIV infection [12]. In this study, we adopt Connell's theory of gender and power as a theoretical framework to explore and clarify the gender dimension in societal beliefs and reactions towards PLWHA and in particular within the family, society and health care system in Port Harcourt, Nigeria.

In Nigeria, power differences exist that are based on gender inequality [3]. Although a matriarchal system co-exists with the patriarchal system in Nigeria [13], often in juxtaposition in the form of matricentric structures [14], patriarchy remains the dominant ideology [15]. Furthermore, a recent United Nations Development report shows that gender inequality in Nigeria is fuelled by socio-cultural practices, patriarchy among other reasons [16]. The patriarchal system in Nigeria uses inheritance customs to place women at a relative disadvantage [3,17], while its structures and processes position women as men's property, perpetuating female subordination [3,18]. These inequalities are associated with inheritance laws that restrict the transfer of property and wealth to males [19]. Women do not inherit land in many cultures in Nigeria [16]. The inheritance laws that favour men place women in economically disadvantaged positions, which make women more vulnerable to infection with HIV/AIDS and its problems. Research carried out among HIV-positive women in Abia state, Nigeria showed that 86.7% of the women in the study were denied rights to family resources [20]. It is also generally accepted that sons provide continuity of the family name in this patriarchal system [19,21,22]. Men are cast as strong-willed and associated with power and leadership; sexual activity is represented as a central marker of masculinity, and is thus portrayed as normal and proper [3,23,24]. Conversely, women are expected to be soft, subservient and gentle [25]. The gender roles in some cases are so well defined that if the males should go into areas meant for females, it is regarded as abomination [25]. Amadiume argues that the traditional gender roles and systems in Nigeria, have been eroded with the changes over time [14], leaving poor women in even more vulnerable positions. While not all cultures have such clearly defined gender roles, many other societies, like those in the Middle East and North Africa, show tolerance of men having multiple partners while encouraging women to practise lifelong fidelity [26]. Furthermore, a study carried out in Namibia shows that people consider men having more than one wife as a right and necessity, while multiple sexual partners are part of the tradition [27]. There is a need to understand socially constructed ideas of gender issues as they relate to sexual behaviour [28] which may help to find solutions for prevention and for the care of PLWHA.

Despite the fact that studies show that there is discrimination against males living with HIV/AIDS [29], and especially against males who have sex with males [30-32], Schwartz and Rutter argue that the punishments for female sexual transgression are swifter, stronger, and more public compared with punishments for males [33]. In a presentation given at the 2004 Bangkok XV International AIDS conference, a female person living with HIV/AIDS from South Africa poignantly highlighted issues of violence and abuse, among others, in her relationship with her partner, following disclosure of her positive HIV status [34]. Another study carried out in Chennai, India, among female sex workers showed that they feared the adverse consequences of disclosure of their positive HIV statuses due to the stigma and discrimination associated with HIV and sex work [35]. Other studies [36-39] have pointed out that gender and power differences affect the stigmatization of PLWHA. In Nigeria, there is a growing amount of literature on HIV/AIDS and gender [40-42]; however, these studies have not considered the gender perspectives that become manifest in differential negative reactions towards PLWHA in society, including in the health care system. Therefore it is important to understand the implications of the dominant male gender norms in Nigerian society as they affect women with respect to HIV/AIDS.

In this paper, our principal objectives are twofold; first, we aim to identify the causes of gender-related differences in societal beliefs and reactions towards men and women living with HIV/AIDS in Port Harcourt, Nigeria. Second, we analyze the impact on the persistence of problems of HIV infection and care for PLWHA in society, including the health care system. To achieve these objectives, we draw on information from the general public, HCPs, and PLWHA and we use Connell's theory of gender and power.

## Methods

A total of 100 in-depth interviews were conducted with 40 people from the general public, 40 HCPs and 20 PLWHA in Port Harcourt, Rivers State, Nigeria. These three different populations coincide with Connell's gender and power theory, which explores gender-related differences at different levels: family level (PLWHA), societal level (the general public) and health care institution level (HCPs). We compared results obtained from the three groups. For the general public, a convenience sample of persons (16 males and 24 females) was used in a multi-venue street-intercept interview technique. A purposive sample of 20 adults living with HIV/AIDS (8 males and 12 females) who were receiving care and counseling from a resource centre in Port Harcourt was also interviewed. The study also used a convenience purposive sample for HCPs. HCPs from five private hospitals and one government resource centre participated in the study. A total of 40 HCPs (20 doctors, 15 nurses, and 5 laboratory scientists; 15 males and 25 females) were interviewed. The street-intercept methodology provides access to segments of the urban population that are hard to reach, and has a high degree of reliability [43,44]. It is also used frequently in studies of sensitive topics such as drug use and sexual behavior [45]. Many of the HCPs interviewed were working concurrently in private and government hospitals, therefore there was some crossover information about their experiences. A semi-structured interview guide was developed on the basis of a literature review in the areas of gender issues, HIV/AIDS and stigma. The study was carried out in the English language. The study was approved by the Rivers State Agency for the control of HIV/AIDS which acted as ethics committee.

### Data Analysis

Interview data were analysed and interpreted using content analysis in which the main ideas were grouped into emerging themes. Nvivo software was used in the analysis. Coding was done by the first author. To check the reliability of coding, an independent researcher coded a random selection of data. When compared, the coding by the first author and the independent researcher showed only a few differences. The few new meanings and discrepancies were discussed and reanalysis of the transcript resulted in fine-tuning of coding and interpretations.

## Results

**Figure 1:**
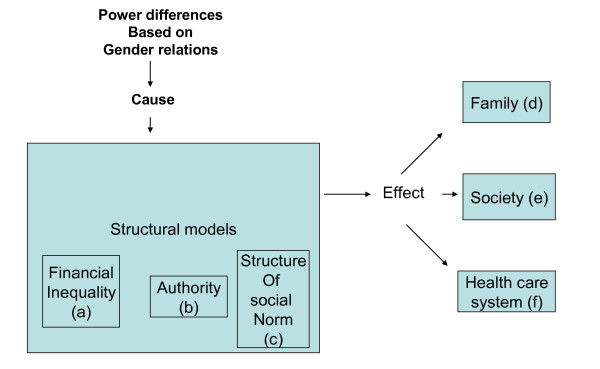
Three part structural model (from Connell's theory of gender and power)

To organise our findings, we grouped the categories of statements according to Connell's theory of gender and power [10]. Figure 1 applies the three-part structural model to our study. The sexual division of labour, the sexual division of power and the structure of affective attachments are represented as financial inequality, authority and the structure of social norms respectively; these are causes of power differences, while the effects of these power differences are apparent at the level of family, society and health care systems. We will first examine the three structures (financial inequality, authority, and structure of social norms) as shown in Figure 1 and how they complement and interact with one another to create power differences that affect the care of and reactions towards male and female PLWHA. Subsequently, we explore the effect of the power differences in our domain of interest at the level of family, society and health care systems.

### Financial inequality

As shown in [a] of Figure 1, one critical aspect of the structural theory regarding gender analysis deals with financial inequality. Financial inequality causes power differences. Our participants mentioned that women often depend on their husbands for money. Women's low financial status makes them even more vulnerable than men. In Nigeria, there are many women who depend on men for financial support and this makes women vulnerable to HIV infection. For instance, a participant from the general public explained about a HIV-positive woman she knows:

*People blame the woman I know with HIV/AIDS. I think the woman got her HIV due to poverty because the woman was not having anybody to care for her, so I think she must have met other men to help her out*. (female, married, business woman).

Even after becoming infected with HIV, women are still more vulnerable because they lack proper financial means to pay for proper care. This vulnerability of women prevents them from questioning their husbands, for instance, when the men are going out with other women:

*Females are a lot more dependent. They depend on their husbands for money to buy drugs, so basically female patients are at a great disadvantage. Most of them are afraid of losing their husbands that are bringing in the money*. (male, married, medical doctor).

A power difference due to the financial inequality between male and female PLWHA is therefore apparent.

#### Authority

Another fundamental part of the structural theory (see [b] in Figure 1) involves control in relationships and authority. There was a clear recognition by the participants that there are power differences in the exercise of authority between men and women in society. Many participants reported that, in Nigeria, men are placed higher than women and this contributes to the manner in which society treats male and female PLWHA. They mentioned that women are always marginalized in one way or another. Either as a cause or as a consequence of the power difference, men have a domineering attitude. Some participants said that a higher level of education and enlightenment reduces the domineering effect; it is worse among uneducated people or those of average education. Men's dominant authority also gives them greater ability to hide things, such as their HIV-positive status, from people - including their wives. In some cases, women who are pregnant find out about their HIV-positive status only when they go to hospital for routine antenatal care or delivery. There are a number of possible explanations why men are able to hide their HIV status. First, men take the decisions in the family and most of the time they do not need the support of their wives before seeking care. Second, wives cannot question men if the men choose not to disclose their HIV status. This participant, who is a female HIV-positive patient, was helpless after discovering her HIV-positive status long after her husband was dead:

*Imagine how my husband hid his HIV status from me so I now have HIV/AIDS from him. If I was a man, I would not be in this condition because I would know myself*. (female, widow, government worker).

In contrast, a woman with HIV may not be able to hide her status because she needs the support of her husband. This has implications for women in terms of becoming infected, seeking early treatment, and their future care.

#### Structure of the social norms

Another basic part of the theory presented (see [c] in Figure 1) involves the structure of social norms. Power differences manifest themselves by the way the social norms are shaped. Our participants mentioned that the man is the head of the family and the woman is the caregiver. Women cook and give the best part of the food to their husbands, even when they are sick. Some participants mentioned that a woman's role is to care for the family and that she does so when her husband is admitted to hospital:

*Here you hardly find any wives who abandon their husbands. Look, here in the male wards, you see their wives by the bedside, but when you go to the female ward, it changes. You hardly see their husbands*. (female, married, nurse).

The social norms prescribe men to go to work and therefore they are not expected to stay by the bedside. Most men need to continue providing for their families even when their wives are sick; in contrast, most married women are housewives, so they are expected to stay in the wards with their sick husbands. Women are observed "managing the situation" because many have no choice other than to stay within the family relation, since most of the time they are dependent on their husbands and like to support their husbands in all possible ways to enable them to recover fast and go back to work. Women protect the family by ensuring that difficult situations are managed. For example, one of the participants from the general public recounted the differences:

*The woman will have more problems at home. If a man has HIV, nobody may hear about it because when he comes home to stay, the wife will not open her mouth to talk about it. She will find a way of managing the situation, but if it is a woman that has it, the first thing the man will do will be to send the woman away from the house, unless it can be proved beyond reasonable doubt that the woman got it from her husband, otherwise there is no remedy for such situation*. (female, married, legal practitioner).

The care-giving role of a woman in the family seems to be a priority concern for female PLWHA even when they are ill:

*It is not the same thing between man and woman with HIV/AIDS because, for instance, when I told this my relative, the first thing she said was, "Oh, what if her husband hears about her HIV status? What about her children?" I do not think a man would react like that. I do not think a man would bother about what his wife's family will think about his HIV status*. (female, married, legal practitioner).

The care-giving responsibilities of women in general sometimes conflict with their own health. For HIV-positive women, the care-giving role becomes complex because they struggle to continue with their role in the family and at the same time deal with problems associated with HIV/AIDS. This affects their ability to seek care in health care institutions.

Although female PLWHA try to care for their families, a medical doctor mentioned that male HIV/AIDS patients have a comparable fear about their social role, that they risk losing their responsibility to be sources of livelihood for the family. This problem was also observed by another health care worker:

*The problem is that some of the HIV patients who are family men care for their family, but their family scatter once they are admitted to hospital. There is now the problem of providing money for the family*. (female, married, nurse).

Men are more concerned with providing income for their families, while women are concerned with efforts aimed at trying to avoid losing their partners. A woman may be sent away by her husband since the woman has left her own family's house to move into the man's family's house, so it is always possible for him to ask her to leave it.

Another important aspect of the structure of social norms, according to our participants, is that there are general beliefs about sexual roles in society. Men are allowed more sexual contacts while women are expected to be faithful. This creates a double standard because, on the one hand, society believes that PLWHA contracted HIV through sex and that therefore they must have been promiscuous, but on the other hand, society allows men more sexual indiscretions while expecting women to keep to one partner. Society also prescribes these female monogamous sexual roles and further accuses HIV-positive women of transgressing the rules and being promiscuous. Many participants expressed the view that females are often viewed as agents of HIV transmission, while men are seen as the victims. These participants from the general public tried to explain how women are looked on as vectors:

*I believe if a woman gets HIV/AIDS it will be worse. People will discriminate against her more than a man. In Nigeria, some people do not believe a man can carry HIV/AIDS; they say it is only women*. (female, single, company worker).

*The person I know with HIV is a woman. It will be different from a man with HIV because people will not think immediately of sex as a cause of the HIV; they will think he got it from the barber's salon*. (female, single, company worker).

Furthermore, a HIV-positive participant mentioned the pressure she experiences with regard to the marriage issue:

*My husband has died from HIV/AIDS and his family and people do not know what killed him. After my husband died, people started asking me why I do not want to get another man. I said in my heart, if only they know what I have, they will not talk to me again*. (female, widow, government worker).

Apparently, people feel that, since her husband is dead, she needs a man to continue giving her support. We will examine how these three structures in Figure 1, which create power differences for male and female PLWHA, affect family, societal and health care systems.

#### Family system

First, as shown in [d] of Figure 1, the effect of power differences manifests itself in the family. This means that women accept whatever situation they find themselves in at home in order to keep their men, who are the source of livelihood, while risking becoming infected with HIV/AIDS by their partners. Additionally, keeping their partners is important for women who are taking anti-retroviral therapy (ART) because they need money to buy the drugs. Other HIV-positive women simply cannot afford the ART:

*I have not started taking antiretroviral treatment because I have no money*. (female, single, office worker).

Another participant who is an HCP mentioned his experience in practice of how a discovery of positive HIV status can affect marriage:

*The sex of the HIV person matters. The few patients I have with a woman HIV-positive and the man HIV-negative, it is hell for the woman, even if they have been sleeping together before the HIV test. As we were trying to console one woman who tested positive for HIV, we looked around for the man; he was gone through the window. They had been sleeping together since six months ago. I was shocked that he could not show a little sympathy*. (male, single, medical doctor).

Sometimes when a woman seems to be sure of herself in knowing that she has been faithful to her husband, she can react differently, as mentioned by this participant who is an HCP:

*Before we wanted to discharge a HIV woman, she said we can call her husband and tell her husband everything about her HIV status. Maybe in her mind she knew she has been faithful to her husband and there was no history of blood transfusion, and if she has that kind of disease, the husband must have given it to her*. (female, married, medical doctor).

Such women emphasize their innocence to gain support from their spouses. Continuity of support from their spouses is very important. Furthermore, a participant who is HIV-positive attributes the negative behaviour towards her by her husband's family to the fact that her husband is not alive:

*They do not even come close to us (me and my children)*. *I am living alone with my children; I am not happy that my husband's family behaved this way. I am suffering with the children- people are running away from us. If it was their brother living, his family may not behave that way*. (female, widow, petty trader).

#### Societal system

At the societal level (see [e] in Figure 1), the effect of power differences can be found. Participants mentioned that the general belief in society is that PLWHA contracted HIV through sexual intercourse which contributes to differences in the way society treats male and female PLWHA. Many participants mentioned that women are expected to avoid premarital sex or remain faithful to their partners, so when they contract HIV, the negative reactions towards them are worse and therefore they are blamed for contracting HIV/AIDS. For instance, this participant who is HIV-positive knows this:

*People will blame me for having HIV because they will say I am a prostitute*. (female, single, office worker).

HIV-positive women are more likely to be judged harshly by society. These participants from the general public share their own understanding of the differences:

*When a girl gets HIV, people think the girl is a flirt and got it through sexual intercourse with a man. If it is a man, the same thing, but most times they say he got it through a haircut. People will not blame the man too much, but for the woman, people blame her and say that she walks about, goes about meeting men. The difference is that they feel women are flirts*. (female, single, company worker).

*It is better to tell government, especially for female people living with HIV/AIDS, because if you do not take care she can spread it to other male people. Some of them know they will die one day so they start flirting about; therefore government should restrict their movement*. (male, married, company worker).

This is a manifestation of the way society shields the men under culturally acceptable excuses while justifying the difference in negative reaction towards PLWHA. Others try to prove their innocence when they contract HIV. There are instances where a female can avoid blame and accusation in the society, but she needs to prove beyond reasonable doubt her innocence in contracting HIV. A possibility of escaping accusation is demonstrated by this quote:

*In very few cases the female is not blamed when the society looks at the person and feels that she is a real Christian and not the type that goes around...but if it is obvious the person got it from sex, the whole family will dump the person. Nobody will listen to her and they will not give the basic help*. (female, married, nurse).

For married people, it is easier for the couple to protect themselves by hiding their HIV statuses, since once the society knows that one of them has HIV, it will automatically be assumed that the other partner has HIV as well. Some participants felt that even if both partners were positive, it was usually only the woman who was blamed by society:

*If a married couple is HIV-positive, the man is kept aside from blame. It is only the woman that will be blamed*. (female, married, nurse).

This also reinforces the more negative reaction towards female PLWHA since society believes that the woman must have given it to the man. Furthermore, even when a man and a woman are both HIV positive, there is a feeling that the woman will experience more problems than the man due to the power difference.

#### Health care system

Third, at the level of the health care system, ([f] in Figure 1) power differences are also shown. The financial dependency of women makes them powerless and that can affect the way that female PLWHA are treated in the hospital by HCPs. This participant, who is an HCP, highlighted the importance of financial considerations when treating female HIV patients:

*One of our reasons for asking the female HIV patients to inform their sexual partner is that in this environment husbands take lots of decisions and if the patient makes demands financially or otherwise on her husband, he may not understand why the doctor does one or two things both in pregnancy and labour. Without telling the husband, the management becomes very difficult for us and I definitely object to that*. (male, married, medical doctor).

In Nigerian society, approval for treatment by female patients' husbands is important because of the assurance it gives to the HCPs that bills will be paid. However, this is a way of allowing gender differences to be perpetuated through the health care system. As a result, the manner in which HCPs approach female HIV patients is different from the way they treat male HIV patients. In general, male HIV patients are not obliged to inform their spouses in order to ensure payment of bills. HCPs enhance the lack of confidentiality in the hospitals for female PLWHA. This participant who is an HCP tried to give an explanation for why women cannot hide their HIV status in comparison with men:

*The woman is different because of societal pressure. If a man has HIV, people do not question him so much, so they can hide it because nobody pressures them to say anything, but when a woman gets HIV, the first thing people or a health care professional will say is, "You should tell your husband or relatives"*. (male, married, medical doctor).

Furthermore, another participant, who is an HCP, emphasized the effect of decision making power of men:

*In Nigeria, this is a man's world. If a woman is HIV- positive, most times you find it difficult to get their husbands to be tested*. (male, married, medical doctor).

These are classic examples of how gender differences are mediated through the health care system because female patients are pressured to inform their significant others, such as their partners. The expectation from society and HCPs for women to disclose to their significant others puts pressure on women, making them helpless and giving them less choice in deciding when to disclose their HIV status and to whom, while giving men the opportunity to decide when to disclose their HIV status and to whom. This also discourages women from seeking care because of their fear for disclosure.

## Discussion

We have shown that Connell's theory of gender and power can explain gender-related power differences, beliefs and reactions towards PLWHA in Port Harcourt Nigeria. Our results show how structures of financial inequality, authority relations and social norms cause power differences between male and female PLWHA. We show how the power differences perpetuate the gender differences in family, society and health care systems and highlight their impact on the persistence of HIV infection and the care of PLWHA. We show that there are similarities and differences in societal beliefs and reactions towards PLWHA between the general public, HCPs and PLWHA. In general, the association of PLWHA with promiscuity was common among the three groups of participants and accusations and blame were directed towards female PLWHA rather than their male counterparts.

Although the existence of power differences was commonly acknowledged among these three groups of participants and in the systems (family, societal and health care), there were, however, differences in the ways that they were shown. The general public mentioned that female PLWHA experienced more negative reactions from society than their male counterparts because the culture marginalises women, not letting them take decisions to prevent infection and to receive timely treatment and care. The HCPs persuade women to inform their partners or relatives of their HIV status, which is a type of difference in the care given to them compared with their male counterparts. For female PLWHA, disclosure of their positive HIV status to their partner or significant other has important implications. On the one hand, disclosure allows female PLWHA to get support from their partners, but on the other hand it may expose them to more risks, such as marital problems at home, especially when such disclosure is a prerequisite before treatment can be given. Moreover, our results already show that female PLWHA were worried about being expelled from the marriage once their husband knew of their HIV status. Power differences also caused some female PLWHA to be unaware of their HIV status until long after their husbands were dead, or until they were in hospital for pregnancy-related reasons. We also found differences between male and female PLWHA in the family system; the general public mentioned that female PLWHA were more concerned about their role in the family; the HCPs noted greater support by women for their husbands in the wards, while female PLWHA were worried about fulfilling this family role. Differences were also observed within marriage, where the general public mentioned that women were more likely to be blamed for contracting HIV when a couple were both HIV-positive.

Our study clearly illustrated how in Nigerian society gender power differences may explain why male PLWHA are in a favoured position over their female counterparts. Our findings also showed how power differences permeate in the family, society and health care system. For instance, in the health care system, our study showed that women who go to hospital are sometimes required to inform their husbands before treatment can be given to them, as a way of guaranteeing the payment of hospital bills. This has implications for the care and support that women receive in the hospitals. There are a number of possible reasons why HCPs need some guarantee of the payment bills before treatment can be started. First, there is no accessible universal health insurance that covers every Nigerian (such coverage would even be more important for women who are vulnerable). In addition, the financial contribution that even those with health insurance will have to make personally for their medical treatment may be unaffordable and thus hinders care. Second, HCPs are aware that many female PLWHA are housewives who are financially dependent on their husbands. In general, the poor economic situation with inequality, higher poverty prevalence for women, the fact that only 47% of women are employed compared with 86% of men and the fact that females in Nigeria have less access to micro credit and grants [16] all contribute to the difference of care given to women compared with their male counterparts. Because health care institutions endeavour to make sure their bills will be paid, they place female PLWHA in vulnerable positions. The financial aspect is particularly important for private hospitals because they have a need to cover their overhead costs, including payment of staff. Although other research shows that women are increasingly taking active decisions on matters affecting their daily lives [46], men are still more domineering and take the main decisions in households [20,47]. In our study, we go further in reporting that men's domineering attitude is worse in families with low socioeconomic status, which impedes women's involvement in family decision-making that is relevant to their health. Other recent research also showed that gender inequalities in health are manifest in traditional practices which attribute women's difficulty in seeking and obtaining adequate care to behavioural lapses by women, such as lack of autonomy by women, leading to lack of decision-making power [47].

Participants in our study reported that men who know they are HIV-positive may hide their status successfully because they do not need support from their wives to go for treatment. Non-disclosure of a positive HIV status has implications for the spread of HIV/AIDS when unprotected sex is practiced. Male PLWHAs' wives almost always protect them and help to prevent their husband's positive status becoming known to others. In contrast, if a woman is HIV-positive, her husband may choose to send her away, exposing the reason behind his actions through informing people of her HIV status.

Our participants emphasized that the link to promiscuity as well as blame comes up immediately once a woman's positive HIV status is disclosed, unlike for her male partner, whose HIV status may be linked to culturally acceptable causes, such as transmission at a barber's salon, allowing him to avoid blame. Our study and others show that HIV-positive women are more likely to be regarded as immoral and to be judged harshly by society [38,48,49]. The measure by which society judges these women who become infected remains outside the control of PLWHA, making them more helpless in defending themselves, especially for those who contracted HIV/AIDS through no fault of their own, such as from their partners. Moral stigma arising from the supposed relation to sex work, stigma tied to the assumption of knowingly infecting men, in addition to the general stigma related to gender, heightens the negative attitude female PLWHA experience [50].

Women who are dependent are unable to seek care in the health care institutions or to procure the anti-retroviral treatment (ART) they need. Our data also show that women are unable to prevent their partners from having sexual relationships with other women since they are dependent on them for financial support. This is in line with a study carried out in Nigeria which shows that women's possibility to confront their husbands' infidelity or to insist on condom use is a highly charged issue (involving- avoidance of pregnancy, counter- accusations and suspicions on both sides) which can threaten the marriage [42].

## Conclusions

Although both men and women suffer the same illness when they contract HIV/AIDS, we argue that society, including HCPs, helps to perpetuate the gender difference in reactions towards PLWHA. The gender difference is embedded in a gendered Nigerian society which favours males and perpetuates power differences; these affect the care that female PLWHA receive, in family, society and health care institutions. Although there have been social changes over time with women gradually becoming more economically self-reliant, the majority are still very dependent on their partners. Women who are financially dependent have less power to take important decisions. They are also unable to seek HIV testing, prevention and early care, and they do not take important decisions that affect their health when they suspect they may be infected with HIV/AIDS, including difficulty bringing their partners for testing. We further point out that health care institutions and HCPs are supposed to apply an egalitarian approach towards caring for PLWHA, but unfortunately they contribute to enhancing the gender difference because of their own need to secure their financial situation (getting paid). Gender-specific power relations and stigma clearly have a great impact on problems related to HIV/AIDS infection and on access to prevention and treatment, which need to be understood. More research is needed to identify the structural and systemic factors that allow gender differences to flourish in society and in the health care systems in order to develop proper policies and programmes for prevention and care.

## Limitations

This study provides important information on the complexities of gender-related power differences that manifests themselves in the family, society and health care institutions. However, there are a number of important limitations that should be considered when interpreting the results. An important limitation in this study is that we employed convenience samples. The street intercept method used for the general public has a disadvantage because it results in a purposeful rather than random sample, limiting external validity. PLWHA were also selectively recruited from a resource centre where many of them had already exposed their positive HIV status. This implies that the information they gave may be different from PLWHA in the general population who are still secretive. Furthermore, we relied on participants' self-reported data. In general, the participants may not have felt free to discuss issues and therefore may have answered or discussed issues in a way they considered was socially desirable. The findings of this study cannot be generalized to other areas of the country, all health care institutions and to other populations but must be interpreted in the context in which the study was done.

## Recommendations

This study has helped to understand gender-related power differences as conceptualized in Connell's theory of gender and power. This understanding can be used for practical purposes in terms of interventions and recommendations. Any political measure that will ameliorate the social situation of women, such as the prevailing financial inequality, authority and social norms, will be beneficial. The government could increase the ability of women to pay for their drugs and increase accessibility to free of charge ART drugs. The government could help to reduce women's vulnerability to increased stigmatization in society by the development and implementation of policies that enhance and protect women's rights, such as equal employment policies. The government should create more job opportunities for women through provision of access to soft loans and micro credit that will help to reduce the financial inequality for women and thereby give them more power and authority. These policies will allow more women to be employed, improve their decision making power in the family and increase women's access to health care. Furthermore, although social norms are informal, specific steps can be taken to protect the vulnerable such as women in society, including in health care institutions. Interventions should be directed at the prevailing societal norms which allow men to take decisions in the home. This can be achieved by appropriate legislation and advocacy at grassroots level with the support of men to counter laws that put women in a disadvantaged position. Problems female PLWHA face in health care institutions can be addressed by creating legislative measures that prevent HCPs from requiring female PLWHA to disclose their HIV status to significant others before starting treatment. It will also help to reduce the differences in reactions female PLWHA experience in health care institutions. Legislation should be enacted to protect women from some of the effects of social norms, such as the sexual roles in society that allow men to have multiple partners and expect monogamy from women. Legislation should further protect women who are sent away by their husbands in ensuring that fair assets are given to them. This will reduce the fear women have of losing their marriage and security. There should be development of a feasible health insurance policy that allows people access to health care and medication. Also, we suggest the government to develop a policy that encourages equality in access to health care for all patients with HIV/AIDS by applying the same conditions to both men and women, and not extending the prevailing social norms to the hospitals. Furthermore, attention should be paid to the training of HCPs to understand the culturally determined gender differences in this respect in order to prevent stigmatizing behaviour and provide effective care for female and male patients with HIV/AIDS.

## Competing interests

The authors declare that they have no competing interests.

## Authors' contributions

NCM conceived of the study, participated in the design, carried out the study, analysis and interpretation of data, drafting of the manuscript for important intellectual content. BVB conceived of the study, participated in the design, analysis and interpretation of data, drafting of the manuscript for important intellectual content and coordination. NDV conceived of the study, participated in the design, analysis and interpretation of data, involved in drafting the manuscript for important intellectual content and coordination. All authors read and approved the final manuscript.

## Pre-publication history

The pre-publication history for this paper can be accessed here:

http://www.biomedcentral.com/1471-2458/10/334/prepub
